# Supplementary material for: Predictors and Risk Score for Immune Checkpoint-Inhibitor-Associated Myocarditis Severity
Source: medRxiv. 2024 Jun 3:2024.06.02.24308336. Preprint. [Version 1] doi: 10.1101/2024.06.02.24308336 (PMC11177901; doi:10.1101/2024.06.02.24308336)

medRxiv preprint doi: https://doi.org/10.1101/2024.06.02.24308336; this version posted June 3, 2024. The copyright holder for this preprint (which was not certified by peer review) is the author/funder, who has granted medRxiv a license to display the preprint in perpetuity.
It is made available under a CC-BY 4.0 International license .

Supplementary Figures Legends

Figure S1: Geographic distribution of study population (discovery cohort)

Figure S2: Univariate (A) and Unimputed (B) (n=238) multivariable models for association with major cardio-myotoxic events.

Abbreviations: CAD: coronary artery disease, CI: confidence interval, COPD: Chronic obstructive pulmonary disease, eGFR: estimated glomerular filtration rate, ICI: immune checkpoint inhibitor, ms: millisecond, mV: millivolt, Ref: reference, ULN: upper limit of normal, VEGFi: Vascular Endothelial Growth Factor Inhibitor, Immunomodulator: non-steroidal immunomodulators only

Figure S3: Spline models. Hazard-ratio for major cardio-myotoxic event at 30 days after presentation as a function of continuous variable predictors using data available at initial presentation. Troponin and creatine kinase were log transformed to account for non-linearity.

Abbreviations: BMI: body mass index, LVEF: left ventricular ejection fraction, CK: creatine kinase, NLR: neutrophil to lymphocyte ratio

Figure S4: Predictors of 30-days mortality with competing risk approach. Illustration of competing risks for study patients (A). Cumulative incidence of competing risks during study period (B). Explanatory multivariable model for association with major cardio-myotoxic events fitted using Aalen Johanssen approach to account for death from other causes (C).

Abbreviations: eGFR: estimated glomerular filtration rate, ICI: immune checkpoint inhibitor, ms: millisecond, mV: millivolt, Ref: reference, ULN: upper limit of normal, VEGFi: Vascular Endothelial Growth Factor Inhibitor, Immunomodulator: non-steroidal immunomodulators only

medRxiv preprint doi: https://doi.org/10.1101/2024.06.02.24308336; this version posted June 3, 2024. The copyright holder for this preprint (which was not certified by peer review) is the author/funder, who has granted medRxiv a license to display the preprint in perpetuity.
It is made available under a CC-BY 4.0 International license .

Figure S5: Harrel's c-index of risk score for association with major cardio-myotoxic events. Graphical and tabular display of c-index when applied on a daily basis to Discovery Cohort (A), Sorbonne University Validation Cohort (B), Mass General Brigham Validation Cohort (C)

Abbreviations: CI: confidence interval

Figure S6: Validation cohorts risk score distribution and cardio-myotoxic event rate. Risk score distribution for Sorbonne University (A) and for Mass General Brigham (B) validation cohorts. Cumulative incidence of major cardio-myotoxic events according to for risk score level in the Sorbonne University (C) and in the Mass General Brigham Hospitals (D) validation cohorts

medRxiv preprint doi: https://doi.org/10.1101/2024.06.02.24308336; this version posted June 3, 2024. The copyright holder for this preprint (which was not certified by peer review) is the author/funder, who has granted medRxiv a license to display the preprint in perpetuity.
It is made available under a CC-BY 4.0 International license .

Supplemental Figures

Table S1: Collaborators by institution (n=757 cases for discovery cohort, n=127 institutions, n=17 countries; and n=149 cases for replication cohorts, n=2 institutions, n=2 countries)

Center	Country	City	Admin	N, discovery cohort	N, validation cohort	Collaborators
Sorbonne University	France	Paris		101	35	Joe Elie Salem, Stephane Ederhy, Sonali Rao, Yves Allenbach, Thomas Similowski
Mass General Brigham	United States	Boston	Massachusetts	1	119	Anju Nohria, Osnat Ithzaki Ben Zadok
University of Texas MD Anderson Cancer Center	United States	Houston	Texas	93	0	Nicolas Palaskas, Anita Deswal
Heidelberg University Hospital	Germany	Heidelberg		36	0	Lorenz Lehmann
Aix-Marseille University. University Mediterranean Center of Cardio-Oncology	France	Marseille		31	0	Jennifer Cautela, Franck Thuny
Hospices Civils de Lyon	France	Lyon		30	0	Pierre-Yves Courand, Anissa Bouali
University of Michigan	United States	Ann Arbor	Michigan	30	0	Salim S. Hayek
Stanford University	United States	Stanford	California	19	0	Han Zhu
University of Texas Southwestern Medical Center	United States	Dallas	Texas	16	0	Vlad G. Zaha
University of Washington	United States	Seattle	Washington	16	0	Richard K. Cheng
Normandie Univ, UNICAEN, INSERM U1086 ANTICIPE; Caen-Normandy University Hospital.	France	Caen		14	0	Joachim Alexandre
CHU Montpellier	France	Montpellier		14	0	François Roubille
Yale School of Medicine	United States	New Haven	Connecticut	14	0	Lauren A. Baldassarre
University of California San Francisco	United States	San Francisco	California	13	0	Javid Moslehi, Alan H. Baik
Tel Aviv Sourasky Medical Center, School of Medicine, Tel Aviv University	Israel	Tel Aviv-Yafo		12	0	Michal Laufer-Perl
Beth Israel Deaconess Medical Center	United States	Boston	Massachusetts	11	0	Aarti Asnani
Maine Medical Center	United States	Portland	Maine	10	0	Sanjeev Francis
University of Virginia	United States	Charlottesville	Virginia	10	0	Elizabeth M. Gaughan
Vanderbilt University Medical Center	United States	Nashville	Tennessee	10	0	Douglas Johnson, Andrew M Hughes
APHP Hopital Lariboisiere	France	Paris		9	0	Guillaume Bailly
Dartmouth Hitchcock Medical Center	United States	Lebanon	New Hampshire	9	0	Danette L Flint
IUHW Mita Hospital	Japan	Tokyo		9	0	Yuichi Tamura
Medical University of Graz; BioTechMed Graz; St. Johann in Tirol General Hospital	Austria	Graz, St. Johann in Tirol		9	0	Peter Rainer, Lisa Moser
APHP Hopital Bichat	France	Paris		8	0	Dimitri Arangalage
CHU Rangueil Toulouse	France	Toulouse		8	0	Eve Cariou
Rabin Medical Center	Israel	Petah Tiqwa		8	0	Osnat Ithzaki Ben Zadok
University of California San Diego Health	United States	San Diego	California	8	0	Anna Narezkina, John Power
University of Utah	United States	Salt Lake	Utah	7	0	Roberta Florido

medRxiv preprint doi: https://doi.org/10.1101/2024.06.02.24308336; this version posted June 3, 2024. The copyright holder for this preprint (which was not certified by peer review) is the author/funder, who has granted medRxiv a license to display the preprint in perpetuity.
It is made available under a CC-BY 4.0 International license .

Center	Country	City	Admin	N, discovery cohort	N, validation cohort	Collaborators
		City				
University of Texas at Austin Ascension Seton	United States	Austin	Texas	7	0	Yan Liu
Barts Health NHS Trust	United Kingdom	London		6	0	Shanthini M Crusz
Maisonneuve-Rosemont Hospital	France	Bordeaux		6	0	Nahema Issa, Maxime Faure
CHU Nantes	France	Nantes		6	0	Nicolas Piriou
Mc Master University	Canada	Hamilton	Ontario	6	0	Darryl Leong, Rocío Baro Vila
Peter MacCallum Cancer Centre	Australia	Melbourne		6	0	Shahneen Sandhu
Disease Unit for Myocarditis and Arrhythmogenic Cardiomyopathies, IRCCS San Raffaele Scientific Institute	Italy	Milan		6	0	Giovanni Peretto
Washington University	United States	St. Louis	Missouri	6	0	Isik Turker, Jesus Jimenez
Hôpital Européen Georges Pompidou, Université Paris Cité	France	Paris		5	0	Assié Eslami
APHP Hopital Henri Mondor	France	Créteil		5	0	Charlotte Fenioux
Bellvitge University Hospital – Catalan Institute of Oncology, IDIBELL, CIBERCV	Spain	Barcelona		5	0	Pedro Moliner
Centre Hospitalier Universitaire Vaudois	Switzerland	Lausanne		5	0	Michel Obeid
Chi Mei Medical Center	Taiwan	Tainan		5	0	Wei Ting Chang
Northwestern University	United States	Chicago	Illinois	5	0	Nausheen Akhter
University of Wisconsin School of Medicine and Public Health	United States	Madison	Wisconsin	5	0	Stephen M. Ewer
Medstar Washington Hospital Center	United States	Washington	District of Columbia	4	0	Seyed Ebrahim Kassaian
University Hospital Erlangen	Germany	Erlangen		4	0	Lucie Heinzerling
University of Pittsburgh Medical Center	United States	Pittsburgh	Pennsylvania	4	0	Joshua E Levenson, Benay Ozbay
CHU Rennes	France	Rennes		3	0	Elise Paven, Elena Galli
Guangdong Academy of Medical Sciences	China	Guangzhou		3	0	
Hospital Universitario La Paz	Spain	Madrid		3	0	Teresa Lopez Fernandez, Lucia Cobarro
Rambam Medical Center	Israel	Haifa		3	0	Manhal Habib
Rosewell Park Cancer Center	United States	Buffalo	New York	3	0	
University of Tsukuba	Japan	Kitaibaraki		3	0	Kazuko Tajiri
Center for Cancer Research National Cancer Institute National Institutes of Health	United States	Bethesda	Maryland	2	0	
CHU Nice	France	Nice		2	0	Fanny Rocher
CHU Tours, University of Tours	France	Tours		2	0	Theodora Bejan-Angoulvant
Cleveland Clinic Foundation	United States	Cleveland	Ohio	2	0	
Emory University	United States	Atlanta	Georgia	2	0	Mehmet Asim Bilen, Susmita Parashar
Houston Methodist Hospital	United States	Houston	Texas	2	0	
Ohio State University Wexner Medical Center	United States	Columbus	Ohio	2	0	Avirup Guha
The First Affiliated Hospital of Weifang Medical University	China	Weifang		2	0	Wenjing Song

medRxiv preprint doi: https://doi.org/10.1101/2024.06.02.24308336; this version posted June 3, 2024. The copyright holder for this preprint (which was not certified by peer review) is the author/funder, who has granted medRxiv a license to display the preprint in perpetuity.
It is made available under a CC-BY 4.0 International license .

Center	Country	City	Admin	N, discovery cohort	N, validation cohort	Collaborators
University Hospital Basel	Switzerland	Basel		2	0	David Koenig, Kirsten Mertz
University of Alabama Birmingham	United States	Birmingham	Alabama	2	0	Carrie Lenneman
University of California at Davis	United States	Davis	California	2	0	
Vidant Medical Center/East Carolina University	United States	Greenville	North Carolina	2	0	
Alfred Health, Monash University	Australia	Melbourne		1	0	Andrew Haydon
Allama Iqbal Medical College	Pakistan	Lahore		1	0	
Anjo Kosei Hospital	Japan	Anjomachi		1	0	
Baylor College of Medicine	United States	Houston	Texas	1	0	
Bern University Hospital	Switzerland	Bern		1	0	
Cedars-Sinai Medical Center Network	United States	Los Angeles	California	1	0	Anja Karlstaedt
Centre Léon Bérard	France	Lyon		1	0	Chloe Lesiuk
Charite Campus Mitte (CMM)	Germany	Berlin		1	0	
CHU Brest	France	Brest		1	0	
CHU Clermont Ferrand	France	Clermont-Ferrand		1	0	Romain Tresorier
CHU Lille	France	Lille		1	0	
Clínica Universidad de Navarra	Spain	Pamplona		1	0	
Cliniques universitaires Saint-Luc	Belgium	Bruxelles		1	0	
Cooper University Hospital	United States	Camden	New Jersey	1	0	
Dana Farber Cancer Institute	United States	Boston	Massachusetts	1	0	
Eisenhower Medical Center	United States	Rancho Mirage	California	1	0	Yazeed Samara
ELK Thorax Center	Germany	Berlin		1	0	Christian Grohe
General Hospital of Chinese People's Liberation Army	China	Beijing		1	0	
Geneva University Hospitals and Medical Faculty	Switzerland	Geneva		1	0	Pierre Yves Dietrich
Hartford Hospital	United States	Hartford	Connecticut	1	0	
Heart Care Centers of Illinois	United States	Chicago	Illinois	1	0	Sean Tierney
Hotel Dieu de France University Hospital	Lebanon	Beirut		1	0	Elie Rassy
ICO Nantes	France	Nantes		1	0	Elvire Mervoyer
Institut Bergonié Centre de Lutte contre le Cancer	France	Bordeaux		1	0	
Japan Community Health Organization Kyushu Hospital	Japan	Fukuoka		1	0	
Keio University	Japan	Tokyo		1	0	Shigeaki Suzuki
Kumamoto University	Japan	Kumamoto		1	0	Satoshi Fukushima
Lahey Hospital and Medical Center	United States	Burlington	Massachusetts	1	0	
Markey Cancer Center, University of Kentucky	United States	Lexington	Kentucky	1	0	
Marshall University	United States	Huntington	West Virginia	1	0	
Mayo Clinic	United States	Rochester	Minnesota	1	0	
Georgia Cancer Center, Augusta University	United States	Augusta	Georgia	1	0	Avirup Guha

medRxiv preprint doi: https://doi.org/10.1101/2024.06.02.24308336; this version posted June 3, 2024. The copyright holder for this preprint (which was not certified by peer review) is the author/funder, who has granted medRxiv a license to display the preprint in perpetuity.
It is made available under a CC-BY 4.0 International license .

Center	Country	City	Admin	N, discovery cohort	N, validation cohort	Collaborators
Memorial Sloan Kettering Weill Cornell Medical College	United States	New York	New York	1	0	
Mitsui Memorial Hospital	Japan	Tokyo		1	0	
Montreal Heart Institute	Canada	Montreal	Quebec	1	0	Maxime Robert-Halabi
Mount Vernon Cancer Centre	United Kingdom	London		1	0	
Nagoya University Graduate School of Medicine	Japan	Nagoya		1	0	Ryota Morimoto
National Cancer Center Hospital East	Japan	Kashiwa		1	0	Kazuko Tajiri
New York Institute of Technology College of Osteopathic Medicine	United States	New York	New York	1	0	
New York-Presbyterian Brooklyn Methodist Hospital	United States	New York	New York	1	0	
Northwell Health	United States	New York	New York	1	0	Robert Copeland-Halperin
Providence St. Joseph Health	United States	Portland	Oregon	1	0	Michael Layoun
Qianfoshan Hospital	China	Jinan		1	0	Jun Wang
Royal North Shore Hospital	Australia	Sydney		1	0	Suran Fernando
Saiseikai Narashino Hospital	Japan	Narashino		1	0	
San Giacomo Hospital	Italy	Monopoli		1	0	Eugenia Rota
Sendai Kousei Hospital	Japan	Sendai		1	0	Yumi Katsume
Shinshu University	Japan	Matsumoto		1	0	Yukiko Kiniwa
St. Luke's International Hospital	Japan	Tokyo		1	0	
Sunnybrook Odette Cancer Centre	Canada	Toronto		1	0	Ellen Warner
Teikyo University School of Medicine	Japan	Tokyo		1	0	Nobuhiko Seki
Tokyo to Saiseikai Central Hospital	Japan	Tokyo		1	0	
Tokyo Women's Medical University	Japan	Tokyo		1	0	
University Hospital LMU	Germany	Munich		1	0	Lucie Heinzerling, Theresa Ruf
University Hospital Mainz	Germany	Mainz		1	0	
University of Chicago	United States	Chicago	Illinois	1	0	
University of Florida	United States	Gainesville	Florida	1	0	Jess DeLaune
University of Massachusetts, Berkshire Medical Centre	United States	Pittsfield	Massachusetts	1	0	
University of North Carolina Medical Center Chapel Hill	United States	Chapel Hill	North Carolina	1	0	
University of southern California	United States	Los Angeles	California	1	0	
University of Toronto	Canada	Toronto		1	0	Nazanin Aghel
VCU Medical center	United States	Richmond	Virginia	1	0	
Veterans Affairs Puget Sound Health Care System-Seattle	United States	Seattle	Washington	1	0	

medRxiv preprint doi: https://doi.org/10.1101/2024.06.02.24308336; this version posted June 3, 2024. The copyright holder for this preprint (which was not certified by peer review) is the author/funder, who has granted medRxiv a license to display the preprint in perpetuity.
It is made available under a CC-BY 4.0 International license .

Table S2: Clinical Characteristics of Validation Cohorts

	Sorbonne University, Paris (France)	Mass General Brigham, Boston (USA)
**Presentation date periods**	April–2023 - April–2024	January–2015 - June 2022
**Sample size**	n=35, prospective	n=119, retrospective[19]
**Age (years, median, (IQR))**	74 (71 - 79)	73 (67 - 76)
**Female (n/N) (%)**	15 (43%)	40 (34%)
**ICI Regimen**		
Anti-PD1 or anti-PDL1 monotherapies	33 (94%)	90 (76%)
Anti-CTLA4 monotherapy	0 (0%)	3 (3%)
Anti-CTLA4 & anti-PD1/PDL1 combination therapy	2 (6%)	26 (22%)
**Cancer type**		
Hemopathy	1 (3%)	2 (2%)
Lung	8 (23%)	30 (25%)
Skin	13 (37%)	36 (30%)
Urogenital	9 (26%)	28 (24%)
Other cancers	4 (11%)	23 (19%)
**Time from 1st ICI infusion to myocarditis (days, median, (IRQ)**	42 (27-79)	52 (26 - 120)
Major Cardiomyotoxic Events (30-days)	11 (31%)	49 (41%)
Respiratory muscle failure requiring ventilation	7 (20%)	6 (5%)
Cardiomyotoxic death	0 (0%)	10 (8%)
Heart Failure requiring intravenous drugs	4 (11%)	30 (25%)
Severe arrhythmia*	5 (14%)	18 (15%)
All-cause mortality (30-days)	0 (0%)	18 (15%)
Risk score parameters within the three first days (day 1 to 3)		
Active thymoma	1 (3%)	0 (0%)
Cardiomuscular symptoms	24 (69%)	117 (98%)
Sokolow Lyon index ≤0.5 mV	3 (9%)	9 (8%)
LVEF<50%	6 (17%)	41 (34%)
Maximum Troponin** (fold upper limit of normal), median (IQR)	26 (10 - 86)	13 (4 - 84)
Diagnostic certainty per updated Bonaca's criteria[5]		
Definite myocarditis	35 (100%)	37 (31%)
Probable myocarditis	0 (0%)	31 (26%)
Possible myocarditis	0 (0%)	51 (43%)

* Severe arrhythmia: defined as sustained ventricular arrythmia, complete heart block, sudden cardiac death, use of atropine or isoproterenol, and/or use of pacemaker/defibrillator
** preferentially cardiac troponin-T, if unavailable cardiac troponin I

medRxiv preprint doi: https://doi.org/10.1101/2024.06.02.24308336; this version posted June 3, 2024. The copyright holder for this preprint
(which was not certified by peer review) is the author/funder, who has granted medRxiv a license to display the preprint in perpetuity.
It is made available under a CC-BY 4.0 International license .

1 **Table S3: Diagnostic properties of the risk score to predict major cardio-myotoxic events at 30 days.**

Risk Score	Sensitivity	Specificity	Positive Predictive Value	Negative Predictive Value
Discovery Cohort: International Redcap				
0+	1.00 (0.98-1.00)	0.00 (0.00-0.01)	0.32 (0.29-0.36)	
1+	0.99 (0.97-1.00)	0.14 (0.11-0.17)	0.35 (0.32-0.39)	0.97 (0.90-1.00)
2+	0.82 (0.77-0.87)	0.48 (0.44-0.52)	0.43 (0.39-0.48)	0.85 (0.80-0.89)
3+	0.46 (0.40-0.53)	0.84 (0.81-0.87)	0.59 (0.52-0.65)	0.77 (0.73-0.80)
4+	0.18 (0.14-0.24)	0.98 (0.96-0.99)	0.80 (0.68-0.89)	0.71 (0.68-0.75)
5+	0.06 (0.03-0.09)	1.00 (0.99-1.00)	0.91 (0.65-0.99)	0.69 (0.65-0.72)
6+	0.01 (0.00-0.04)	1.00 (0.99-1.00)	0.96 (0.35-0.99)	0.68 (0.64-0.71)
Validation Cohort: Sorbonne University				
0	1.00 (0.70-1.00)	0.00 (0.00-0.16)	0.31 (0.18-0.48)	
1	1.00 (0.70-1.00)	0.29 (0.15-0.49)	0.39 (0.24-0.58)	1.00 (0.60-1.00)
2	0.82 (0.51-0.96)	0.67 (0.47-0.82)	0.53 (0.31-0.74)	0.89 (0.66-0.98)
3	0.36 (0.15-0.65)	0.92 (0.73-0.99)	0.67 (0.30-0.91)	0.76 (0.58-0.88)
4	0.18 (0.04-0.49)	0.96 (0.78-1.00)	0.67 (0.20-0.94)	0.72 (0.54-0.85)
5	0.00 (0.00-0.30)	1.00 (0.84-1.00)		0.69 (0.52-0.82)
6	0.00 (0.00-0.30)	1.00 (0.84-1.00)		0.69 (0.52-0.82)
Validation Cohort: Mass General Brigham				
0	1.00 (0.91-1.00)	0.00 (0.00-0.06)	0.41 (0.33-0.50)	
1	1.00 (0.91-1.00)	0.01 (0.00-0.08)	0.42 (0.33-0.51)	1.00 (0.17-1.00)
2	0.80 (0.66-0.89)	0.50 (0.39-0.61)	0.53 (0.41-0.64)	0.78 (0.64-0.88)
3	0.29 (0.18-0.43)	0.90 (0.80-0.95)	0.67 (0.45-0.83)	0.64 (0.54-0.73)
4	0.02 (0.00-0.12)	0.99 (0.92-1.00)	0.50 (0.09-0.91)	0.59 (0.50-0.67)
5	0.00 (0.00-0.09)	1.00 (0.94-1.00)		0.59 (0.50-0.67)
6	0.00 (0.00-0.09)	1.00 (0.94-1.00)		0.59 (0.50-0.67)

Figure S1: Geographic distribution of study population (Discovery Cohort)

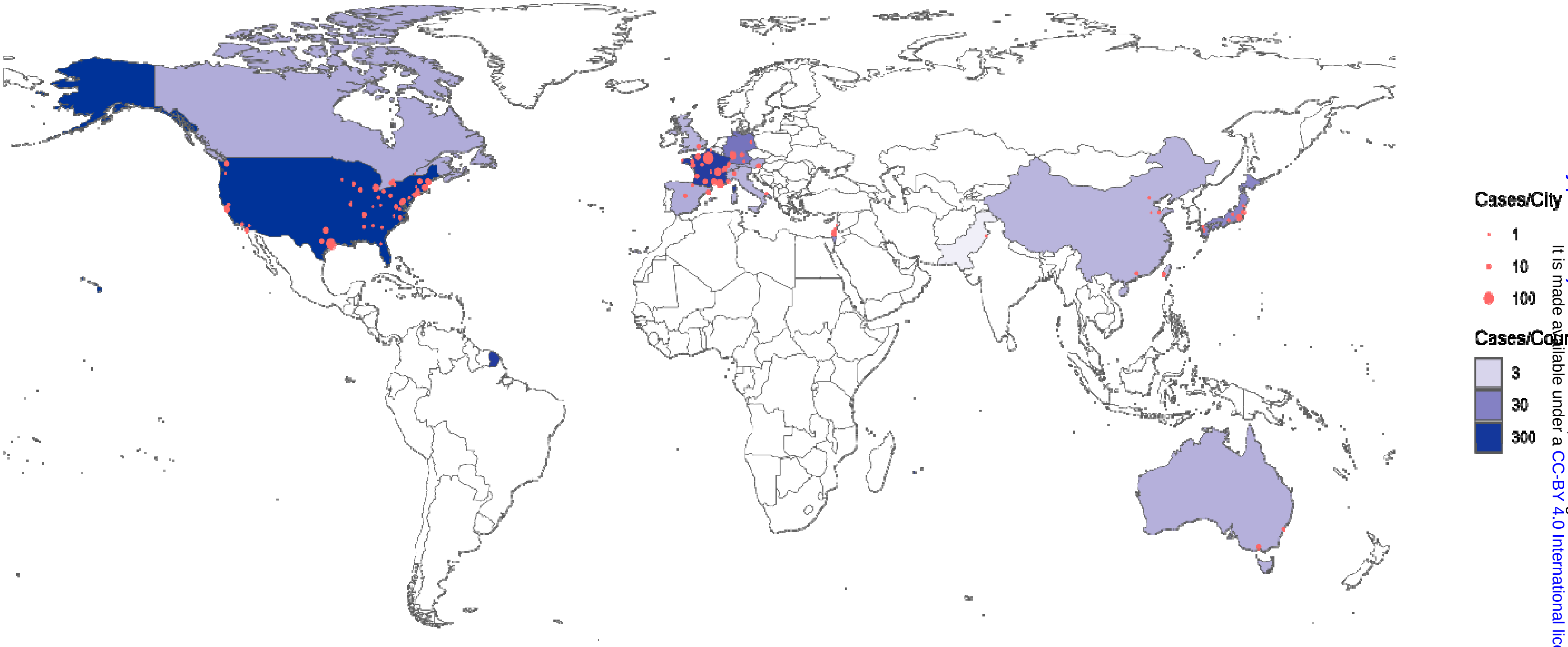

medRxiv preprint doi: https://doi.org/10.1101/2024.06.02.24308336; this version posted June 3, 2024. The copyright holder for this preprint (which was not certified by peer review) is the author/funder, who has granted medRxiv a license to display the preprint in perpetuity.
It is made available under a CC-BY 4.0 International license .

medRxiv preprint doi: https://doi.org/10.1101/2024.06.02.24308336; this version posted June 3, 2024. The copyright holder for this preprint (which was not certified by peer review) is the author/funder, who has granted medRxiv a license to display the preprint in perpetuity.
It is made available under a CC-BY 4.0 International license .

Figure S2: Univariate (A) and Unimputed (B) (n=238) multivariable models for association with major cardio-myotoxic events.

A.

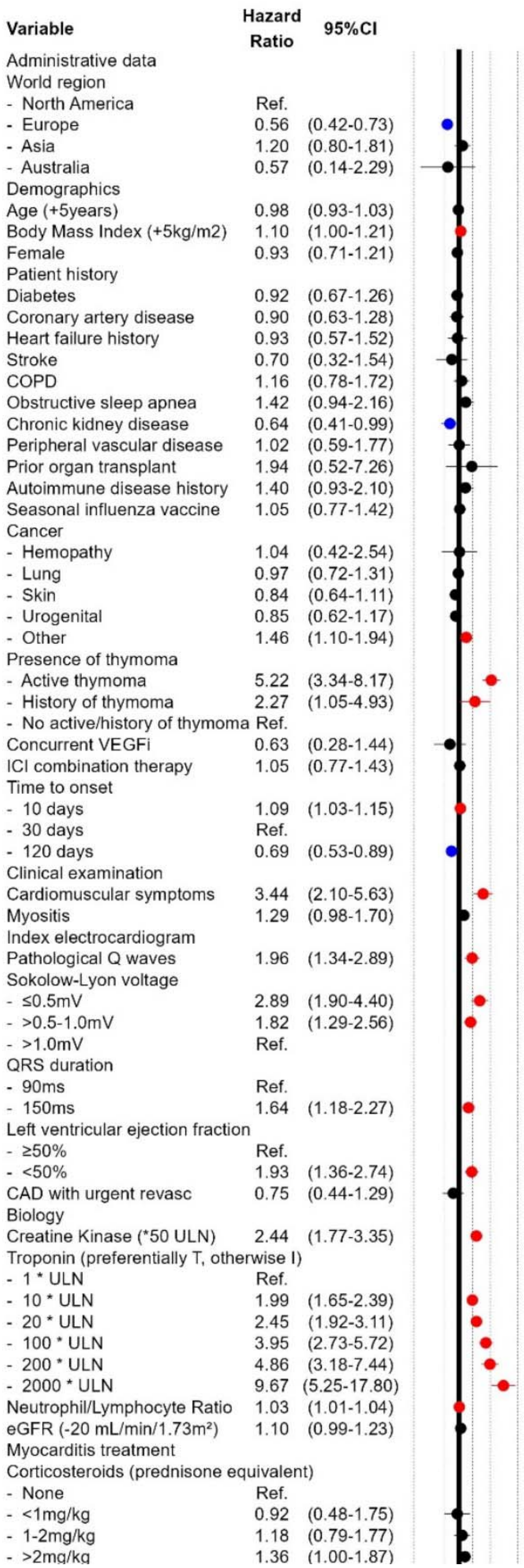

B.

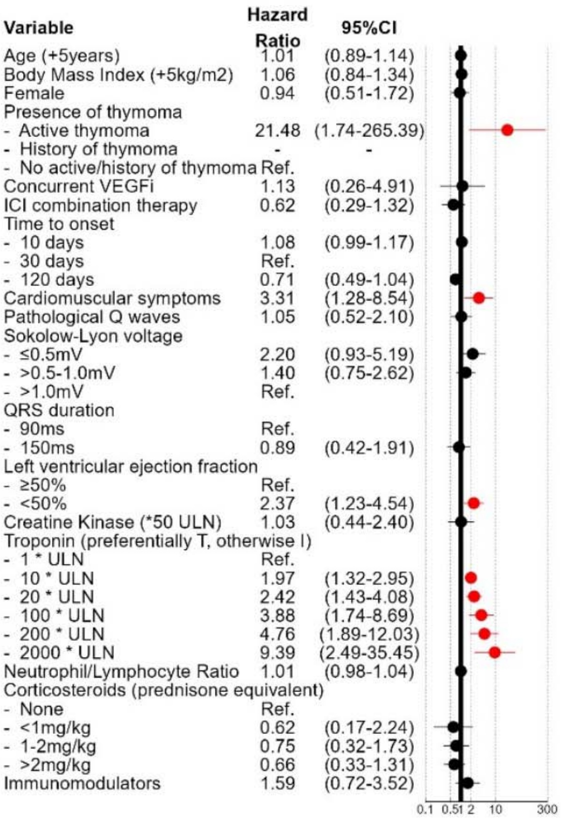

medRxiv preprint doi: https://doi.org/10.1101/2024.06.02.24308336; this version posted June 3, 2024. The copyright holder for this preprint (which was not certified by peer review) is the author/funder, who has granted medRxiv a license to display the preprint in perpetuity.
It is made available under a CC-BY 4.0 International license .

<u>Abbreviations</u>: CAD: coronary artery disease, CI: confidence interval, COPD: Chronic obstructive pulmonary disease, eGFR: estimated glomerular filtration rate, ICI: immune checkpoint inhibitor, ms: millisecond, mV: millivolt, Ref: reference, ULN: upper limit of normal, VEGFi: Vascular Endothelial Growth Factor Inhibitor, Immunomodulator: non-steroidal immunomodulators only

Figure S3: Spline models. Hazard-ratio for major cardio-myotoxic event at 30 days after presentation as a function of continuous variable predictors using data available at initial presentation. Troponin and creatine kinase were log transformed to account for non-linearity. w

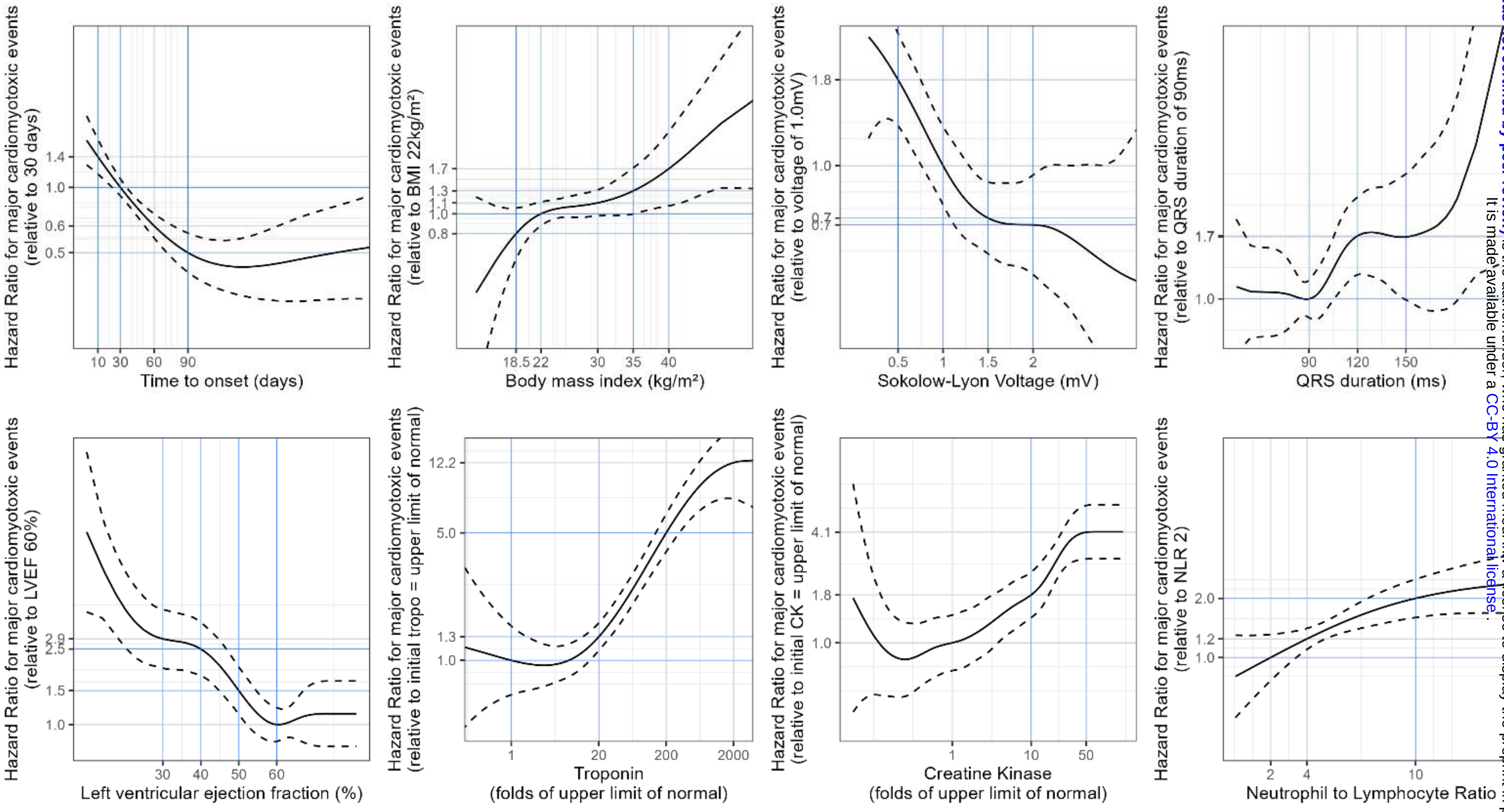

Abbreviations: BMI: body mass index, LVEF: left ventricular ejection fraction, CK: creatine kinase, NLR: neutrophil to lymphocyte ratio

medRxiv preprint doi: https://doi.org/10.1101/2024.06.02.24308336; this version posted June 3, 2024. The copyright holder for this preprint (which was not certified by peer review) is the author/funder, who has granted medRxiv a license to display the preprint in perpetuity.
It is made available under a CC-BY 4.0 International license .

medRxiv preprint doi: https://doi.org/10.1101/2024.06.02.24308336; this version posted June 3, 2024. The copyright holder for this preprint (which was not certified by peer review) is the author/funder, who has granted medRxiv a license to display the preprint in perpetuity.
It is made available under a CC-BY 4.0 International license .

Figure S4: Predictors of 30-days mortality with competing risk approach. Illustration of competing risks for study patients (A). Cumulative incidence of competing risks during study period (B). Explanatory multivariable model for association with major cardio-myotoxic events fitted using Aalen Johanssen approach to account for death from other causes (C).

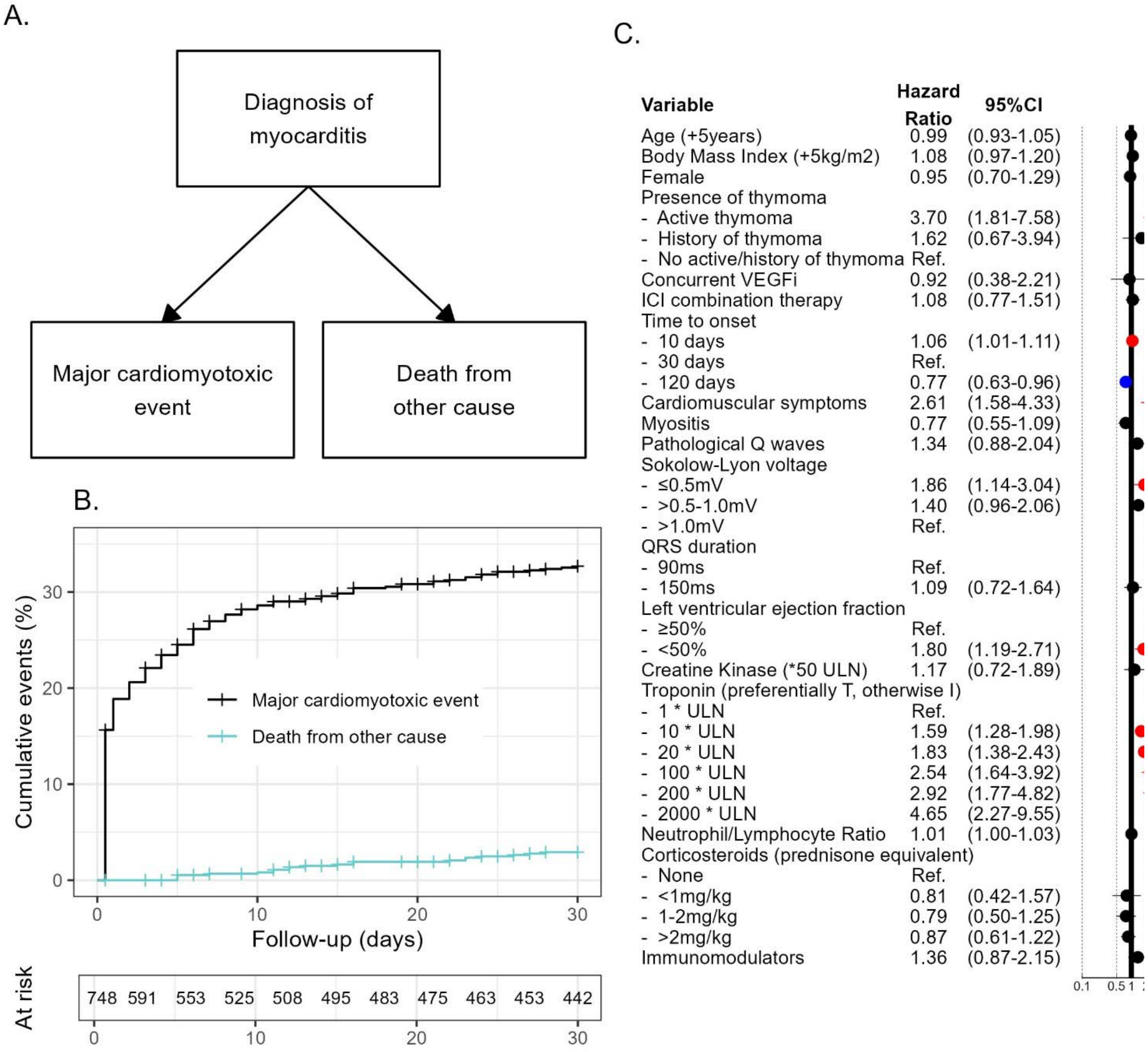

Abbreviations: eGFR: estimated glomerular filtration rate, ICI: immune checkpoint inhibitor, ms: millisecond, mV: millivolt, Ref: reference, ULN: upper limit of normal, VEGFi: Vascular Endothelial Growth Factor Inhibitor, Immunomodulator: non-steroidal immunomodulators only

medRxiv preprint doi: https://doi.org/10.1101/2024.06.02.24308336; this version posted June 3, 2024. The copyright holder for this preprint (which was not certified by peer review) is the author/funder, who has granted medRxiv a license to display the preprint in perpetuity.
It is made available under a CC-BY 4.0 International license .

Figure S5: Harrel's c-index of risk score for association with major cardio-myotoxic events. Graphical and tabular display of c-index when applied on a daily basis to Discovery Cohort (A), Sorbonne University Validation Cohort (B), Mass General Brigham Validation Cohort (C)

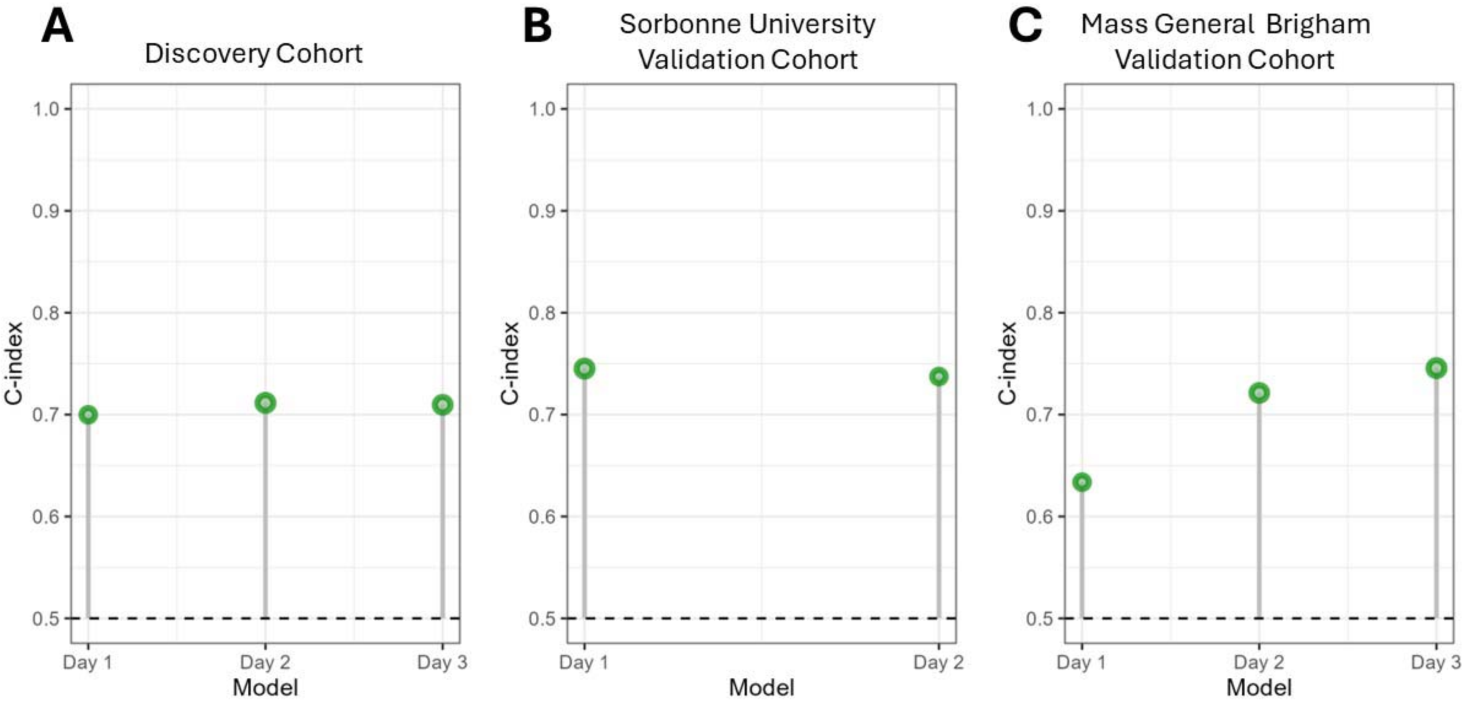

	C-index (95%CI)
Day 1	0.700 (0.667-0.730)
Day 2	0.711 (0.662-0.755)
Day 3	0.709 (0.659-0.754)

	C-index (95%CI)	N Cases
Day 1	0.745 (0.534-0.855)	35
Day 2	0.737 (0.518-0.870)	30

	C-index (95%CI)	N Cases
Day 1	0.634 (0.556-0.709)	119
Day 2	0.721 (0.623-0.798)	102
Day 3	0.746 (0.650-0.820)	93

Abbreviations: CI: confidence interval

medRxiv preprint doi: https://doi.org/10.1101/2024.06.02.24308336; this version posted June 3, 2024. The copyright holder for this preprint
(which was not certified by peer review) is the author/funder, who has granted medRxiv a license to display the preprint in perpetuity.
It is made available under a CC-BY 4.0 International license .

Figure S6: Validation cohorts risk score distribution and cardio-myotoxic event rate. Risk score distribution for Sorbonne University (A) and for Mass General Brigham (B) validation cohorts. Cumulative incidence of major cardio-myotoxic events according to for risk score level in the Sorbonne University (C) and in the Mass General Brigham University (D) validation cohorts

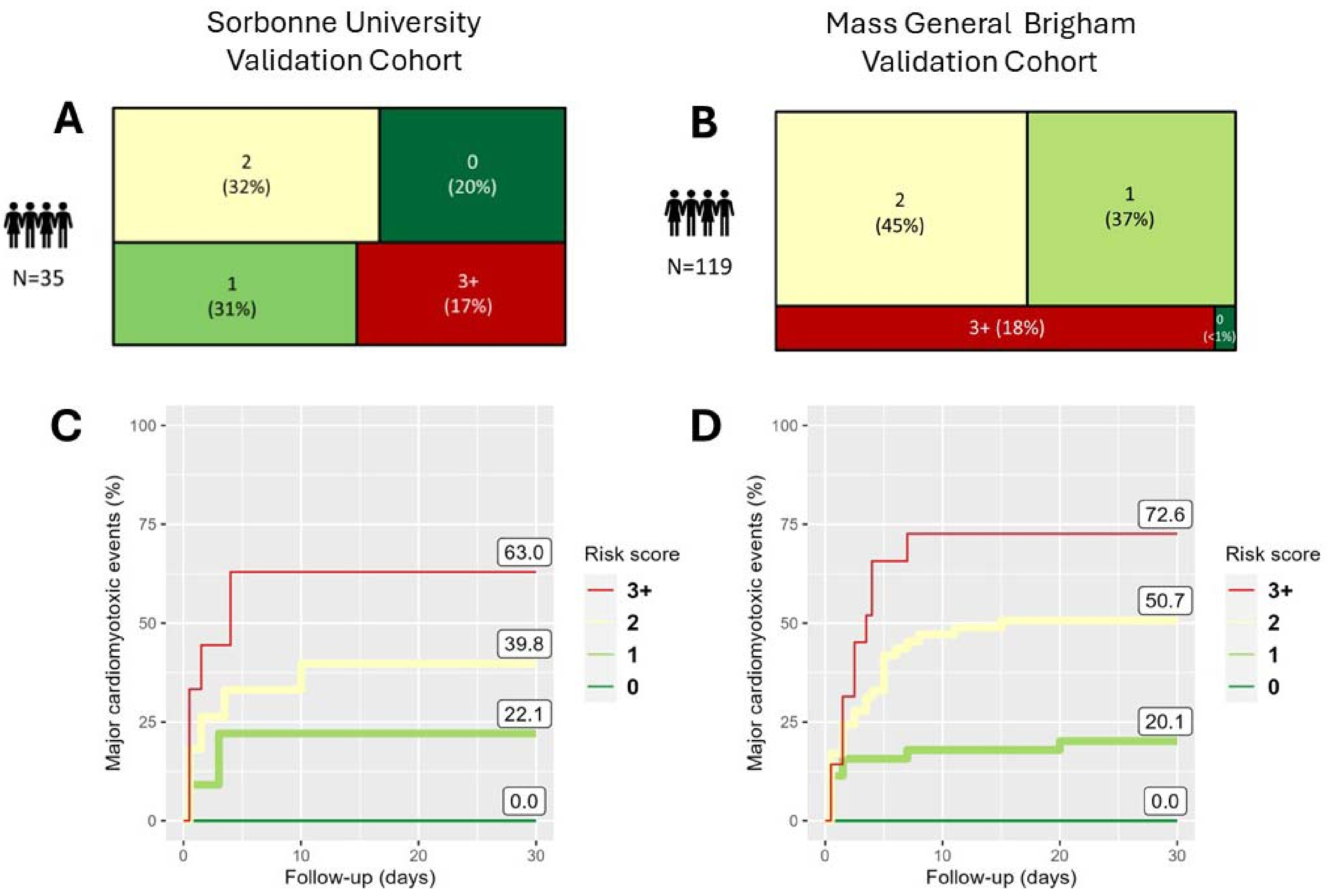

Supplement: Supplement 1 [file NIHPP2024.06.02.24308336v1-supplement-1.pdf]
